# Septin Mutations in Human Cancers

**DOI:** 10.3389/fcell.2016.00122

**Published:** 2016-11-09

**Authors:** Dimitrios Angelis, Elias T. Spiliotis

**Affiliations:** Department of Biology, Drexel UniversityPhiladelphia, PA, USA

**Keywords:** septins, cancer, neoplasia, missense mutations, tumorigenesis, oncogenes, tumor suppressors, Ras GTPases

## Abstract

Septins are GTP-binding proteins that are evolutionarily and structurally related to the *RAS* oncogenes. Septin expression levels are altered in many cancers and new advances point to how abnormal septin expression may contribute to the progression of cancer. In contrast to the *RAS* GTPases, which are frequently mutated and actively promote tumorigenesis, little is known about the occurrence and role of septin mutations in human cancers. Here, we review septin missense mutations that are currently in the Catalog of Somatic Mutations in Cancer (COSMIC) database. The majority of septin mutations occur in tumors of the large intestine, skin, endometrium and stomach. Over 25% of the annotated mutations in SEPT2, SEPT4, and SEPT9 belong to large intestine tumors. From all septins, SEPT9 and SEPT14 exhibit the highest mutation frequencies in skin, stomach and large intestine cancers. While septin mutations occur with frequencies lower than 3%, recurring mutations in several invariant and highly conserved amino acids are found across different septin paralogs and tumor types. Interestingly, a significant number of these mutations occur in the GTP-binding pocket and septin dimerization interfaces. Future studies may determine how these somatic mutations affect septin structure and function, whether they contribute to the progression of specific cancers and if they could serve as tumor-specific biomarkers.

Septins are GTP-binding proteins that form higher order filamentous structures, which function primarily in the spatial organization and compartmentalization of many cellular processes (Caudron and Barral, 2009; Mostowy and Cossart, 2012; Spiliotis and Gladfelter, 2012). In human cells, septins comprise a family of 13 paralogous genes, which encode 13 different septins (SEPT1-SEPT12, SEPT14) with multiple isoform variants (Kinoshita, 2003b; Pan et al., 2007; Russell and Hall, 2011). Evolutionarily, septins belong to the same class of GTPases as the *RAS* oncogenes (Leipe et al., 2002). Similar to the Ras proteins, the core GTP-binding structure of septins consists of alternating α-helices, β-sheets and loops (P-loops) that come in contact with the phosphate groups of GTP (Leipe et al., 2002). Septins, however, contain additional helices (α0, α5′, and α6) and β-strands (e.g., β7, β8), which in part support tandem dimerization into oligomers and polymers (Sirajuddin et al., 2007, 2009; Macedo et al., 2013). Assembly of these septin heteromers depends on GTP-binding and hydrolysis, which further stabilizes the dimerization interfaces through allosteric effects (McMurray, 2014; Zent and Wittinghofer, 2014; Zeraik et al., 2014). In contrast to the monomeric Ras GTPases, whose function relies on the hydrolysis and exchange of GTP by GTPase activating proteins (GAPs) and guanine exchange factors (GEFs), septins hydrolyze and exchange GTP on their own, albeit at very slow rates (Sheffield et al., 2003; Vrabioiu et al., 2004; Huang et al., 2006; Abbey et al., 2016).

Based on sequence similarity, mammalian septins are categorized in four groups: SEPT2 (septins 1, 2, 4, and 5), SEPT3 (septins 3, 9, and 12), SEPT6 (septins 6, 8, 10, 11, 14) and SEPT7 (Kinoshita, 2003a; Cao et al., 2007; Pan et al., 2007). Due to lack of a critical threonine residue, which coordinates the hydrolysis of GTP, septins of the SEPT6 group are thought to bind GTP constitutively (Sirajuddin et al., 2009; Zent and Wittinghofer, 2014). Septins vary mainly in the N- and C-terminal sequences that flank the GTP-binding domain. SEPT7 and septins of the SEPT6 group contain α-helical coiled-coil domains in their C-terminal tails, while SEPT9 contains an elongated N-terminal tail, which is enriched with prolines and interacts with microtubules and actin microfilaments (Bai et al., 2013; Smith et al., 2015). With the exception of the SEPT6 group, all septins contain a polybasic domain, which has been shown to interact with membrane phosphoinositides (Zhang et al., 1999; Casamayor and Snyder, 2003).

Septins assemble into oligomers and polymers in a combinatorial fashion, forming complexes that include a septin from each one of the four groups (Kinoshita, 2003a; Nakahira et al., 2010; Sandrock et al., 2011). Through their GTP-binding domains, septins dimerize in tandem to form a non-polar hetero-octamer of a 2:2:2:2 stoichiometry (Kim et al., 2011; Sellin et al., 2011). This palindromic dimer of two SEPT2/6/7/9 complexes is posited to be the basic unit of most mammalian septin heteromers; septins of the same group substitute one another within the SEP2/6/7/9 complex. The expression of certain septins, however, varies significantly between different tissues and organs, and the exact composition of septin complexes is not well known (Hall et al., 2005; Connolly et al., 2011a; Sellin et al., 2014). Moreover, septin heteromers of alternative compositions or stoichiometries have been reported, suggesting that the SEPT2/6/7/9 mode of assembly might not be a panacea (Dolat et al., 2014a).

Septins interface functionally with molecular mechanisms that underlie the membrane- and cytoskeleton-based processes of the cell. Named after their roles in partitioning the membranes of the two emerging daughter cells in late mitosis, septins initially became known for their functions in cytokinesis (Longtine et al., 1996; Kinoshita and Noda, 2001; Joo et al., 2005; McMurray and Thorner, 2009). Over time, membrane-associated septins were discovered to maintain diffusion barriers, controlling protein localization, and to modulate exocytic membrane fusion (Kartmann and Roth, 2001; Caudron and Barral, 2009; Bridges and Gladfelter, 2015). Recently, septins were found to affect mitochondrial division (Pagliuso et al., 2016; Sirianni et al., 2016), pointing to as-yet-unknown roles in the biogenesis of membranous organelles. Of note, septins have been implicated in the biogenesis of multi-vesicular bodies and autophagosomes, as well as in lysosomal homeostasis (Mostowy et al., 2010; Traikov et al., 2014; Dolat and Spiliotis, 2016).

In the cytoplasm, septins associate with the actomyosin and microtubule cytoskeletons. The organization and contractile properties of actin-myosin filaments are modulated by septins, which cross-link and bend actin filaments into functional structures such as the cytokinetic contractile ring, the actin stress fibers that power cell migration, and cellular protrusions such as filopodia, pseudopodia and lamellipodia (Kinoshita et al., 1997, 2002; Joo et al., 2007; Kremer et al., 2007; Shankar et al., 2010; Hu et al., 2012; Mizutani et al., 2013; Dolat et al., 2014b; Mavrakis et al., 2014). Similarly, septins affect the organization, dynamics and post-translational modifications of the microtubule cytoskeleton, impacting the morphogenesis of epithelia and neurons (Spiliotis et al., 2008; Bowen et al., 2011; Ageta-Ishihara et al., 2013; Bai et al., 2013; Froidevaux-Klipfel et al., 2015). Moreover, microtubule-associated septins are essential for proper chromosome alignment and segregation during mitosis, and the cytoskeleton-dependent transport of membrane vesicles in interphase cells (Spiliotis et al., 2005, 2008; Bai et al., 2016).

As our knowledge of septin functions continues to expand, it is becoming increasingly evident that abnormalities in septin expression have a major impact on cellular homeostasis and human health. To date, septins are linked to various disease states including neurodegenerative, neuromuscular and blood disorders as well as infertility and developmental disabilities (Dolat et al., 2014a; Marttinen et al., 2015). Notably, septin expression levels are widely altered in almost every cancer type from leukemias and epithelial carcinomas to melanomas and gliomas (Cerveira et al., 2011; Connolly et al., 2011a; Dolat et al., 2014a).

## Alterations of septin expression in cancer

Historically, SEPT9 was the first septin implicated in cancer. In the late 1990s/early 2000s, three independent lines of evidence linked SEPT9 to cancer. First, SEPT9 was identified as a fusion partner of the mixed lineage leukemia (MLL) gene (Osaka et al., 1999), which translocates to various chromosomal loci, giving rise to chimeras that promote the oncogenic potential of MLL; subsequently more septins were identified as MLL fusion partners (Cerveira et al., 2011). Second, the murine SL-3 retrovirus that causes T-cell lymphomas was found to preferentially integrate into the *SEPT9* gene locus (Sorensen et al., 2000). This observation was reminiscent of the seminal discovery of proto-oncogenes, which trigger cancerous growth after insertion of retroviral DNA into the host genome (Hayward et al., 1981; Payne et al., 1982). Third, the human SEPT9 gene was mapped to the chromosomal locus 17q25.3, which is frequently deleted in sporadic ovarian and breast cancers (Kalikin et al., 2000; Russell et al., 2000). Multiple copies of the SEPT9 gene, however, were also found in a variety of human tumors and similar amplifications of the SEPT9 gene were observed in mouse models of breast cancer (Montagna et al., 2003).

**Figure 1:**
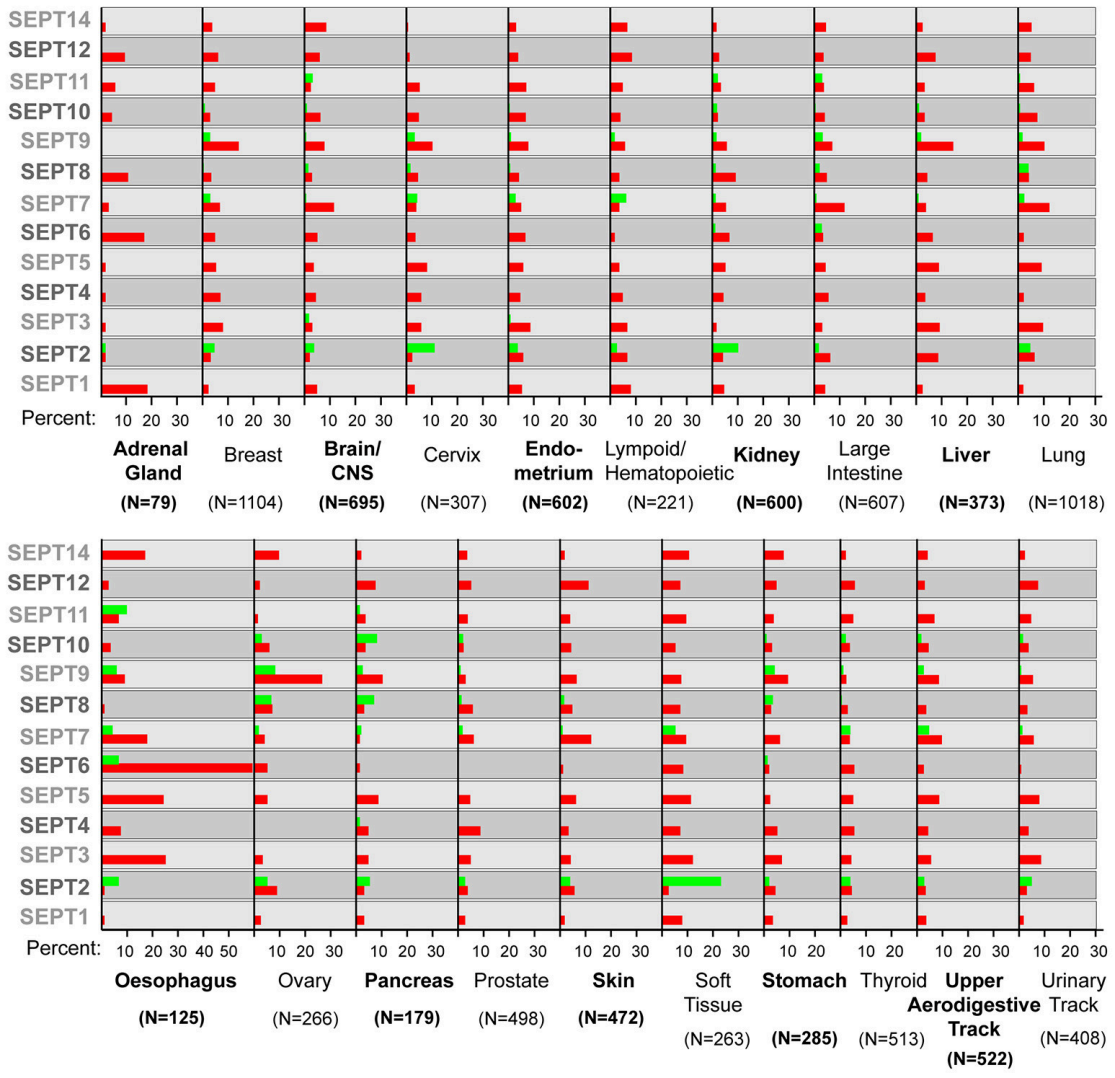
**Septin over- and under-expression in human cancers**. Bar graphs show the percentage of tumor samples, in which a specific septin is over-expressed (red) or under-expressed (green). In the COSMIC database, the thresh-hold value for over- or under-expression is set at two times the standard deviation from the mean values of expression in tumor samples that were diploid for a septin gene. The number (N) of samples analyzed is shown in parenthesis under each tumor type. Note that several septins are both over- and under-expressed in samples of the same tumor type.

Following these early findings, an increasing number of studies began to report the over-expression and down-regulation of specific septins in a variety of hematological malignancies and solid tumors. In addition to the MLL-septin chimeras, the SEPT4/ARTS isoform was found to be down-regulated in acute lymphoblastic leukemia (ALL) and genetic ablation of SEPT4 increased the levels of hematopoietic stem cells, which became resistant to apoptosis (Larisch et al., 2000; Elhasid et al., 2004; Garcia-Fernandez et al., 2010). Alterations in septin expression have been reported in brain tumors (glioblastomas), skin (squamous cell carcinomas, melanomas), kidney (renal cell carcinomas), colorectal, lung and hormonally regulated cancers such as prostate, breast, ovarian and endometrial cancers (Hall and Russell, 2004; Dolat et al., 2014a; Montagna et al., 2015). In the vast majority of these cancers, septins are over-expressed (Figure 1), but occasional down-regulation, ectopic expression and epigenetic alterations have also been reported (Tanaka et al., 2001; Liu et al., 2010; Payne, 2010; Connolly et al., 2011b; Shen et al., 2012; Montagna et al., 2015). Based on these abnormalities, diagnostic tests were developed for urothelial and colorectal cancers, which screen respectively for the expression of the Bradeion isoform of SEPT4 in the urine and the methylation of the SEPT9 gene in the blood (Tanaka et al., 2001, 2003; Grutzmann et al., 2008; Warren et al., 2011; Bongiovanni et al., 2012).

## Emerging roles of septins in cancer

In contrast to classical tumor suppressors and oncogenes, which induce cancer by loss and gain of function mutations, septins are thought to belong to a broader class of cancer genes that affect tumorigenesis as a consequence of altered levels of expression (Sager, 1997; Hall and Russell, 2004). Given that septins function as hetero-oligomers, it is unclear how up- or down-regulation of a septin isoform could contribute to cancer progression. However, a growing number of studies show that abnormalities in the expression levels of a single septin bestow cellular properties akin to tumorigenic phenotypes (Gonzalez et al., 2009; Garcia-Fernandez et al., 2010; Connolly et al., 2011b; Dolat et al., 2014b). Moreover, decreasing the expression of a single septin has been shown to suppress tumor growth *in vivo* (Tanaka et al., 2002; Yu et al., 2016).

Taken together with advances in the cell biological functions of septins, it is now evident that septins are linked to the molecular mechanisms that underlie hallmarks of cancer such as resistance to cell death, proliferation, angiogenesis and invasion and metastasis. The ARTS isoform of SEPT4 (SEPT4_i1) is regarded as a pro-apoptotic tumor suppressor, whose down-regulation in leukemia could render resistance to pro-apoptotic stimuli (Gottfried et al., 2004; Garcia-Fernandez et al., 2010). SEPT9_i1 binds and prevents the degradation of the c-Jun-N-terminal kinase (JNK), which promotes tumor cell proliferation (Gonzalez et al., 2009). Similarly, septins have also been reported to suppress the degradation of the epidermal growth factor receptor (EGFR) and the receptor-protein tyrosine kinase Erb2/HER2, which are linked to signaling pathways that trigger cell proliferation and migration (Diesenberg et al., 2015; Marcus et al., 2016). Notably, SEPT9_i3 is phosphorylated by the cell cyclin-dependent kinase 1 (Cdk1), which controls entry into mitosis and promotes cell proliferation and survival (Liu et al., 2008; Estey et al., 2013).

Sustained proliferation requires a metabolic reprogramming, which includes the internalization of carbon sources and amino acids from the interstitial fluid by macropinocytosis (Bloomfield and Kay, 2016). Ras mutants have been known to upregulate macropinocytosis and septins were recently found to affect the intracellular transport of macropinosomes in a dose dependent manner (Bryant et al., 2014; Dolat and Spiliotis, 2016). Oxygen supply is equally important for the metabolic demands of tumors, which induce angiogenesis under hypoxic conditions (Hoff and Machado, 2012). SEPT9_i1 binds and facilitates the nuclear import of the hypoxia-inducible factor-1α (HIF1α), whose transcriptional activity promotes angiogenesis (Amir et al., 2009; Golan and Mabjeesh, 2013).

Multiple studies have shown that over-expression of SEPT9 isoforms enhances cell motility and invasion, and suggest that SEPT9 upregulation is programmed by the epithelial-to-mesenchymal transition (EMT) that drives the development of carcinomas (Chacko et al., 2005; Shankar et al., 2010; Connolly et al., 2011b; Fuchtbauer et al., 2011; Dolat et al., 2014b). SEPT2 and SEPT7 have also been implicated in the migration and invasion of breast cancer and glioblastoma cells (Jiang et al., 2014; Zhang et al., 2016). While SEPT2/7 promote the cell migration and invasion of breast cancer cells, SEPT7 appears to have the opposite role in gliomas (Jiang et al., 2014; Zhang et al., 2016). Over-expression of other septins such as SEPT1 has been suggested to promote the migration of skin cancer cells (Mizutani et al., 2013).

In addition to these roles, which are intimately associated with the hallmarks of cancer, septins may contribute to the genomic instability of cancer, resistance to anti-cancer drugs and the promotion of cell growth by the tumor microenvironment. Alterations in septin expression affect chromosome alignment and segregation as well as cytokinesis, which may result in the loss or gain of whole chromosomes (Spiliotis et al., 2005; Estey et al., 2010; Thompson et al., 2010; Menon et al., 2014). Indeed, over-expression of SEPT9_i1 in human mammary epithelial cells increases aneuploidy, which correlates with defects in centrosome duplication, chromosome segregation and cytokinesis (Peterson et al., 2011). In cancer cell lines, elevated expression of SEPT9 isoforms (e.g., SEPT9_i1/i4) and down-regulation of SEPT10 have been reported to confer resistance to the anti-cancer microtubule-targeting drug paclitaxel (Amir and Mabjeesh, 2007; Froidevaux-Klipfel et al., 2011; Chacko et al., 2012; Xu et al., 2012). Future studies will determine whether dysregulation of septin expression affects cancer resistance *in vivo*. Interestingly, a recent study showed that *in vivo* septins are required for the remodeling of the extracellular matrix by cancer-associated fibroblasts (CAFs). In addition, septins partly promote CAF-induced tumor growth and pro-angiogenic activity (Calvo et al., 2015). This is the first evidence of septins playing a role in the organization and properties of the tumor microenvironment, which is important for the progression of cancer.

## A global view of septin missense mutations in human cancers

In contrast to the alterations in septin expression, which have been extensively documented in a variety of cancers, septin mutations remain virtually undocumented. The Catalog of Somatic Mutations In Cancer (COSMIC; http://cancer.sanger.ac.uk/cosmic) is an on-line database that compiles cancer genomic information from scientific publications, The Cancer Genome Atlas (TCGA) and the International Cancer Genome Consortium (ICGC) databases. Currently, COSMIC contains data for 42 different primary tumor types (e.g., intestinal, skin, kidney), which were derived from approximately 1.23 million patients. The majority of these samples are from patients with haemotopoietic and lymphoid cancers (~32%). Large intestine, lung and thyroid cancer samples account for ~14%, ~13%, and ~5% of patients, respectively. All other tumor types are each under 5% of the total pool of patients. Presently, COSMIC contains data from screening a cumulative of 28,611 genes, including isoforms from identical genes, and 301,848 unique mutations are catalogd.

**Figure 2:**
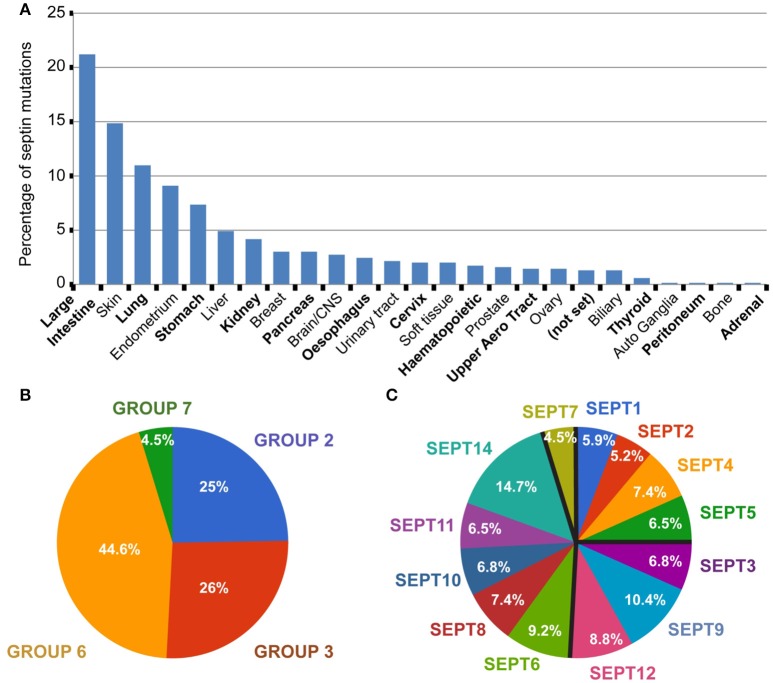
**Global view of septin missense mutations in human cancers. (A)** All 693 septin missense mutations were grouped per tumor type. Bar graph shows the percentage of total septin mutations that occur in each tumor type. Tumors of uncertain type are designated as “not set.” **(B,C)** Pie charts show the percentage of septin mutations per septin group **(B)** and individual septin paralog **(C)**.

In COSMIC, 693 septin missense mutations are reported; for this review's purpose, we focused on single amino acid substitutions and not on gene deletions, duplications, translocations or epigenetic alterations. A number of these septin mutations are found in different tumor types or different patients with the same type of tumor. Over 20% of the septin mutations correspond to large intestine tumors (Figure 2A). Approximately 15% of septin mutations belong to skin cancers, >10% to lung tumors and >5% to endometrial and stomach tumors. The percentage of total septin mutations for any other tumor type is lower than 5%. Given that intestine, skin, endometrial and stomach cancers represent 14.4%, 3.3%, 1.3%, and 2% of the total tumor samples, respectively, the percentage of septin mutations in these tumor types is above the relative abundance of samples. Similarly, the percentage (4–5%) of septin mutations in liver and kidney tumors is more than double the percentage (1.3–1.4%) of these tumors in the total samples screened.

Septins of the SEPT6 group account for 44.6% of total septin mutations, while the SEPT2 and SEPT3 groups each harbor ~25% of septin mutations (Figure 2B). From all 13 septin paralogs, the top two most mutated septins are SEPT14 and SEPT9, which contain ~15% and ~10% of total mutations, respectively (Figure 2C). Each of the remaining septin paralogs account for 5–9% of total mutations. Interestingly, the ubiquitously expressed SEPT7 is the least mutated, accounting for less than 5% of total mutations (Figures 2B,C).

**Figure 3:**
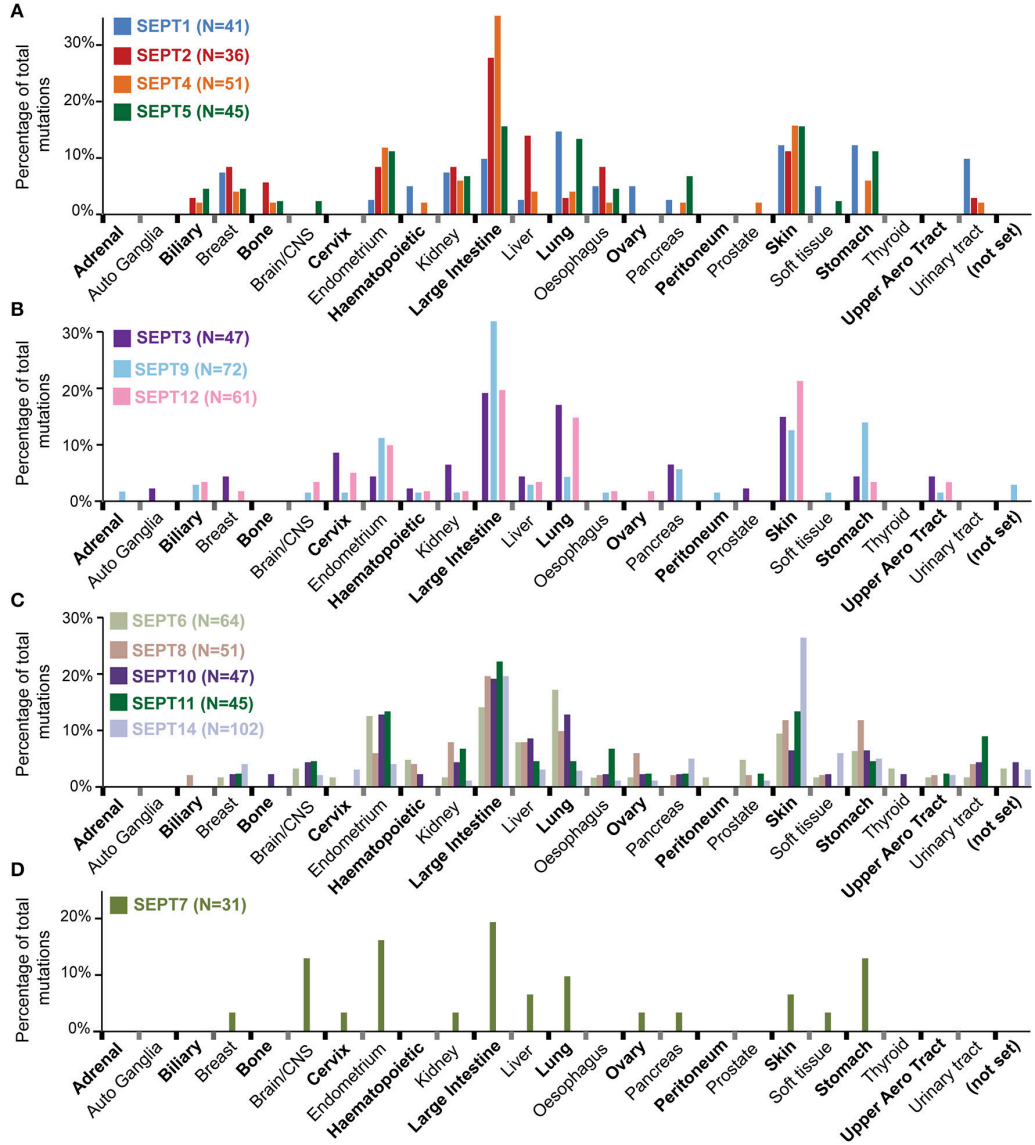
**Distribution of septin mutations by tumor type**. Bar graphs show the distribution of missense mutations across tumor types for the individual septin paralogs of the SEPT2 **(A)**, SEPT3 **(B)**, SEPT6 **(C)**, and SEPT7 **(D)** groups. Septin paralog-specific mutations were binned under each tumor type and the percentages of total mutations per each tumor type were derived and graphed. The *N* values shown in parenthesis correspond to the number missense mutations identified in COSMIC for the corresponding septin paralogs. Tumors of uncertain identity are designated as “not set.”

Grouping the mutations of each septin paralog under their corresponding tumor types shows that 25–35% of the SEPT2, SEPT4, and SEPT9 mutations are found in large intestine tumors (Figures 3A,B). SEPT2 and SEPT9 had notably more mutations (~14%) in liver and stomach cancers, respectively, relative to all other septins (Figures 3A,B). The majority of SEPT12 and SEPT14 mutations occur in skin (20–26%), large intestine (20%) and lung (13–15%) cancers (Figures 3B,C). Similar trends are observed for SEPT3, SEPT5, SEPT6, SEPT10, and SEPT11. Compared to all other septins, SEPT7 has a higher percentage of mutations in cancers of the central nervous system (~13%) and endometrium (~16%) (Figure 3D).

In contrast to the high mutation frequencies of Ras GTPases and other classical oncogenes (Fernandez-Medarde and Santos, 2011; Pylayeva-Gupta et al., 2011; Chang et al., 2016), the incidence of septin mutations appear to be below 3% (Table 1). Among the top ten most frequent septin mutations, SEPT14 and SEPT9 top the list with frequencies of 2.56 and 1.86% in skin and stomach cancers, respectively (Table 1). The same septins also have the highest frequencies (1.3–1.5%) in tumors of the large intestine. The mutation frequencies (1.25%) of SEPT9 and SEPT6 in endometrial tumors, and of SEPT3 in cervical cancer are also among the highest of all septins. Overall, septin mutations occur with the highest frequency in cancers of the skin, large intestine, endometrium and stomach.

**Table 1:** **Septins with the highest mutation frequencies ***(f)*****.

**Rank**	**SEPT**	**Primary tumor**	***f* (%)**	**Samples screened**	**Total samples**
1^*^	SEPT14	Skin	2.56	1094	16,706
2	SEPT9	Stomach	1.86	592	24,308
3	SEPT9	Large intestine	1.55	1482	179,020
4	SEPT14	Large intestine	1.35	1482	179,020
5	SEPT9	Endometrium	1.25	640	16,706
–	SEPT6	Endometrium	1.25	640	16,706
7	SEPT3	Cervix	1.24	322	5828
8	SEPT4	Large Intestine	1.21	1482	179,020
9	SEPT12	Skin	1.10	1094	40,749
10	SEPT14	Soft tissue	1.06	567	40,032

* *SEPT6 frequency in peritoneal tumors is 10%, but only 10 out of 400 samples have been screened*.

## Recurring mutations in conserved septin residues and domains

Ras proteins are known for containing highly conserved amino acids that are mutated with high frequencies in a variety of cancers (Fernandez-Medarde and Santos, 2011; Pylayeva-Gupta et al., 2011). These mutational hotspots comprise amino acids, which are key for the hydrolysis of GTP, and have been developed as prognostic markers and targets for anti-cancer therapies (Pylayeva-Gupta et al., 2011; Chang et al., 2016; Lu et al., 2016).

**Figure 4:**
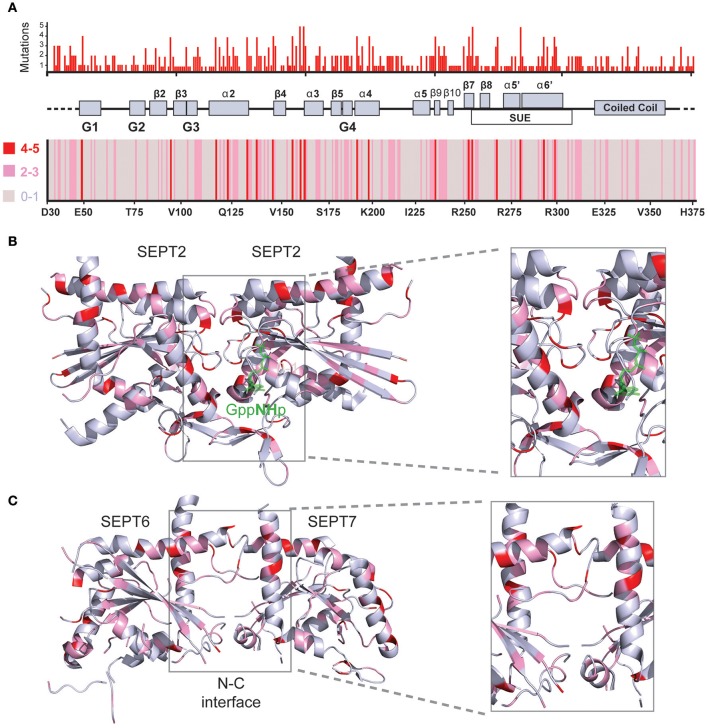
**Structural mapping of septin missense mutations. (A)** The amino acid sequences of all 13 human septins were aligned with the Clustal Omega program (EMBL-EBI) and amino acid positions were numbered starting with amino acid 30 of SEPT6. Histogram (top) shows the cumulative number of missense mutations that occur for each amino acid position across all septin paralogs. A heat-map (bottom) representation indicates the number of mutations for each amino acid position by color-coding 0-1 mutations in gray, 2-3 mutations in pink and 4-5 mutations in red. A schematic diagram depicts the secondary structure elements and domains that correspond to the amino acid positions on the x-axis of the histogram and heat-map. On the x-axis of the heatmap, the amino acid identities and numbers of SEPT6 are shown for reference purposes. **(B,C)** Ribbon cartoons of the 3D crystal structure of SEPT2 (**B**; PDB code: 3FTQ) and the SEPT6/7 dimer **(C)** from the crystal structure of SEPT2/6/7 (PDB code: 2QAG) depict the location and cumulative number of mutations shown in the histogram and heatmap above. Gray areas contain none or one mutation, while pink and red areas harbor 2-3 and 4-5 mutations, respectively. Insets show magnified views of the GTP-binding pocket and G-G interface **(B)** as well as the N-C dimerization interface of SEPT6/7 **(C)**. Ribbon cartoons were generated with the PyMOL software and amino acid locations were also color-coded in PyMOL.

Aligning the sequences of the 13 septin paralogs and scoring all amino acids with missense mutations reveals that there are fully conserved residues with multiple mutations (Figure 4A, Table 2). In Figure 4A, the positions of the most frequently mutated amino acids are identified by a color-coded heatmap, which indicates the cumulative number of mutations for each amino acid position. Interestingly, several amino acids that contain multiple mutations are positioned in the GTP-binding pocket, the septin unique element and the G-G as well as the N-C dimerization interfaces of septins (Figures 4B,C).

**Table 2:** **Missense mutations in fully conserved amino acids across all septins**.

	**Polybasic**		β**1 sheet**		……….		α**1 helix**				……….		β**3 sheet**		**Switch II**			α**2 helix**		……….	
SEPT1				G32E			S39C								G92S				E121K	R126Q	
SEPT2			F38L	G44S															E133K		
SEPT3																					R170H
SEPT4						G156D							L204F						E240K		
SEPT5																G113W					
SEPT6									L62R			V93M		T98M							
SEPT7			F52L								E103D										
SEPT8					G54D														E135V		R149M
SEPT9									L318I			V354I								R399C R399H	
SEPT10																		E151K			R171H
SEPT11		F40L										V92A					D111N			R137C R137H	
SEPT12				G56V				T64M		F70L F70C	E101D						D118E				
SEPT14	G50R			G59R		G64R G64E	S66L					V103I				G116D		E137Q		R148C	R157H R157C R157L

	β**4 sheet**		…….		α**3 helix**	β**5 sheet**	**G4 (AKAD)**		…….	…….			β**7 sheet**	β**8 sheet**	………		α**5**′ **helix**		α**6 helix**		
SEPT1										P224R											
SEPT2								D185A		P236S		G241A									R300C
SEPT3								D210N D210E		P260S	V263L		R280Q								
SEPT4			G264S									G348D									R408L
SEPT5		F159V													E273K						
SEPT6	C151F	F154L				P181S			Y213C	P234L										T290I	R299H
SEPT7					D177H		A197S														
SEPT8						P183H							R256Q								R301W
SEPT9			G419D						Y473F										R537W		
SEPT10			G183D	L186V									R278H	G283V							
SEPT11																N264Y	F270V	L273P			
SEPT12											V249M		R266L R266W R266Q								
SEPT14							A196V					G249E	R264H						R285Q	T300A	

## Mutations in the GTP-binding pocket of septins

**Figure 5:**
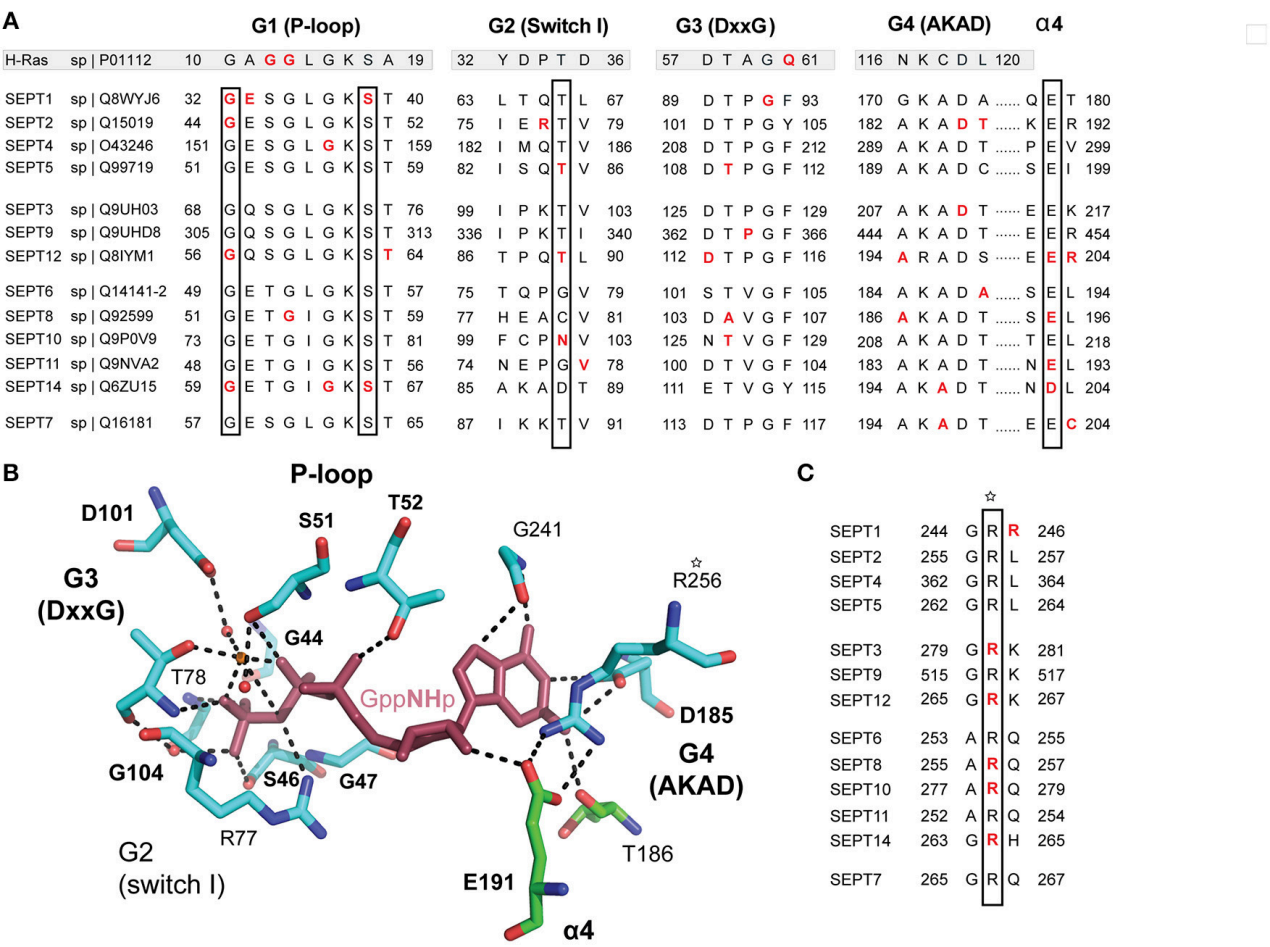
**Cancer mutations in conserved residues of the septin GTP-binding pocket. (A)** Sequence alignment of the G1 (P-loop), G2, G3, and G4 motifs of all 13 human septins. The corresponding sequences of H-Ras are shown in shaded boxes. The H-Ras amino acids in codons 12, 13, and 61 (highlighted in red font) are sites of highly frequent activating mutations among all *RAS* oncogenes. Septin residues with missense mutations are shown in red font. The positions of highly conserved amino acids with three or more missense mutations across all septins are outlined with rectangle boxes. **(B)** Stick cartoon of the atomic structure of the GTP-binding pocket of SEPT2 (PDB code: 3FTQ) shows the position of highly conserved amino acids, which make contact with the GTP analog GppNHp (maroon) and are mutated in several human cancers. Dashed lines outline interactions between the side chains of amino acids and GppNHp. The atomic representation and bonds (dashed lines) were generated in PyMOL using the “sticks mode” and “measure distance” functions of the software. **(C)** Sequence alignment shows an invariant arginine (asterisk), which is mutated in five different septins and makes contact with the ribose moiety of GTP. This amino acid is also involved in the polar interactions of the G-G interface (Figure 6) and is positioned at the beginning of the septin unique element.

The GTP-binding pocket of septins comprises the G1 (Walker A) motif GxxxxGKS/T, which forms the P-loop that interacts with the phosphate residues of the GTP, the G4 motif AKAD, which interacts with the guanine base of GTP, and the G3 motif DxxG, which binds Mg^2+^ and is essential for the coordination of GTP hydrolysis (Figure 5A). The GTP-binding pocket also contains a threonine, which is analogous to the Thr-35 of the G2 sequence of the Ras GTPases (Sirajuddin et al., 2009). In conjunction with the glycine of the G3 motif, this threonine (T78 in SEPT2) is critical for the hydrolysis of GTP and is present in all septins with the exception of those of the SEPT6 group, which are thought to bind GTP constitutively (Sirajuddin et al., 2009; Zent et al., 2011; Macedo et al., 2013; Zent and Wittinghofer, 2014). In addition, an invariant arginine residue, which is positioned in the beginning of the septin unique element, and a highly conserved glutamate, which is located in the α4 helix following the G4 motif, make contact with the guanine base of GTP (Figures 5B,C).

In Ras GTPases, the conserved residues G12 and G13 are heavily mutated in human cancers (Pylayeva-Gupta et al., 2011). These residues correspond to the third and fourth amino acids of the G1 Walker A motif GxxxxGKS/T (Figure 5A). In septins, only G13 is conserved as an invariant glycine and is mutated only once in SEPT8 (Figure 5A). Mutation of this residue in SEPT7 has been previously shown to disrupt GTP-binding and septin dimerization (Abbey et al., 2016). The first invariant glycine of the GxxxxGKS/T motif is mutated in four different septins (SEPT1, SEPT2, SEPT12, and SEPT14) and the invariant serine, which has been shown to affect SEPT7 oligomerization (Abbey et al., 2016), is mutated in SEPT1 and SEPT14.

Mutations in the highly conserved threonine (T78 in SEPT2; G2 sequence), which is responsible for GTP hydrolysis in septins, occur only in SEPT5 and SEPT12. Interestingly, the threonine of SEPT12 (T89) is mutated into methionine, which is the same mutation identified in infertile male patients with asthenoteratozoospermia (Kuo et al., 2012). This mutation is found in a cancer of the central nervous system. Of note, an arginine residue (R198 in SEPT2) that is highly conserved among all septins with the catalytic threonine (T78 in SEPT2) is mutated in four septins. This arginine is located in the α4 helix, which underlies the GTP-binding pocket.

In the guanine-binding AKAD motif (G4), six mutations are identified in three different amino acids. The invariant alanine in the first position of the AKAD motif is mutated in SEPT2 and SEPT8. The aspartate residue is mutated once in SEPT2 and twice in SEPT3. Remarkably, a mutation in the same amino acid of SEPT12 (D197N) is linked to male infertility and has been shown to disrupt SEPT12 assembly into functional filaments (Kuo et al., 2012). Moreover, the aspartate of the AKAD motif is also the site of a temperature-sensitive mutation (D182N) in the Cdc10 septin of the budding yeast *S. cerevisiae* (Weems et al., 2014). Interestingly, this missense mutation has been shown to switch the nucleotide specificity of p21Ras from guanosine to xanthosine, and is likely to result in a nucleotide-free septin (Schmidt et al., 1996).

The G3 motif DxxG harbors six mutations (Figure 5A), but the conserved D and G residues are only mutated once in SEPT12 and SEPT1, respectively. The remaining four mutations occur in the second and third positions of the DxxG motif; three mutations occur in a highly conserved threonine. Among all residues of the GTP-binding pocket, the invariant guanine-interacting arginine of the SUE (R256 in SEPT2) and glutamate of α4 helix (E191 in SEPT2) are the most heavily mutated (Figures 5A,C). Interestingly, an invariant glycine (G241 in SEPT2), which is positioned between the β9 and β11 sheets and interacts with guanine, is also mutated in three different septins (Figure 5B). Notably, this glycine marks a dominant negative temperature-sensitive mutation (G247E) in the yeast septin Cdc12 (Weems et al., 2014).

Overall, the mutational pattern of the septin G domain does not exhibit the high frequency and amino acid selectivity of Ras GTPases. Given that Ras mutational hotspots such as the G12 and G13 residues of the P-loop are involved in the mechanism of GTP hydrolysis by GTPase-activating proteins (GAPs), this difference may be due to the slow and mechanistically unique GTPase activity of septins, which do not involve GAPs and depend functionally more on hetero-oligomerization than GTP turnover. Thus, activating mutations in the G domain of Ras GTPases could be more self-selective in the evolution of cancer. It is nevertheless evident that a significant number of mutations are clustered in the GTP-binding pocket of septins and involve several invariant residues that come in contact with both the guanine base and phosphates of GTP.

## Mutations in the G-G and N-C dimerization interfaces of septins

Septins assemble into functional higher order structures by homo- and hetero-dimerization, which is structurally mediated by their GTP-binding domains (Sirajuddin et al., 2007; Kim et al., 2012). The X-ray crystallographic structures of several septin homodimers (e.g., SEPT2, SEPT7, SEPT3) and of the heterotrimeric SEPT2/6/7 have revealed two dimerization interfaces, which are termed G-G and N-C interfaces (Sirajuddin et al., 2007, 2009; Zent et al., 2011; Macedo et al., 2013). The G-G interface involves elements of the GTP-binding pocket including the loop regions between the antiparallel β7 and β8 strands, which are a unique features of the septin G-domain compared to Ras proteins, and the loop regions between the β4 strand and α3 helix (Sirajuddin et al., 2007, 2009; Zent et al., 2011; Macedo et al., 2013). The N-C interface is mediated by interactions between the N- and C-terminal regions of the GTP-binding domains, and is characterized by an upper and lower half. The upper N-C interface involves the N-terminal helix α2 and the C-terminal helix α6 of the SUE domain (Sirajuddin et al., 2007, 2009; Zent and Wittinghofer, 2014). The lower N-C interface is supported by interactions between residues of the N-terminal α0 helices and between amino acids of the C-terminal β2/β3 sheets and N-terminal α0 helix (Sirajuddin et al., 2009; Zent and Wittinghofer, 2014).

**Figure 6:**
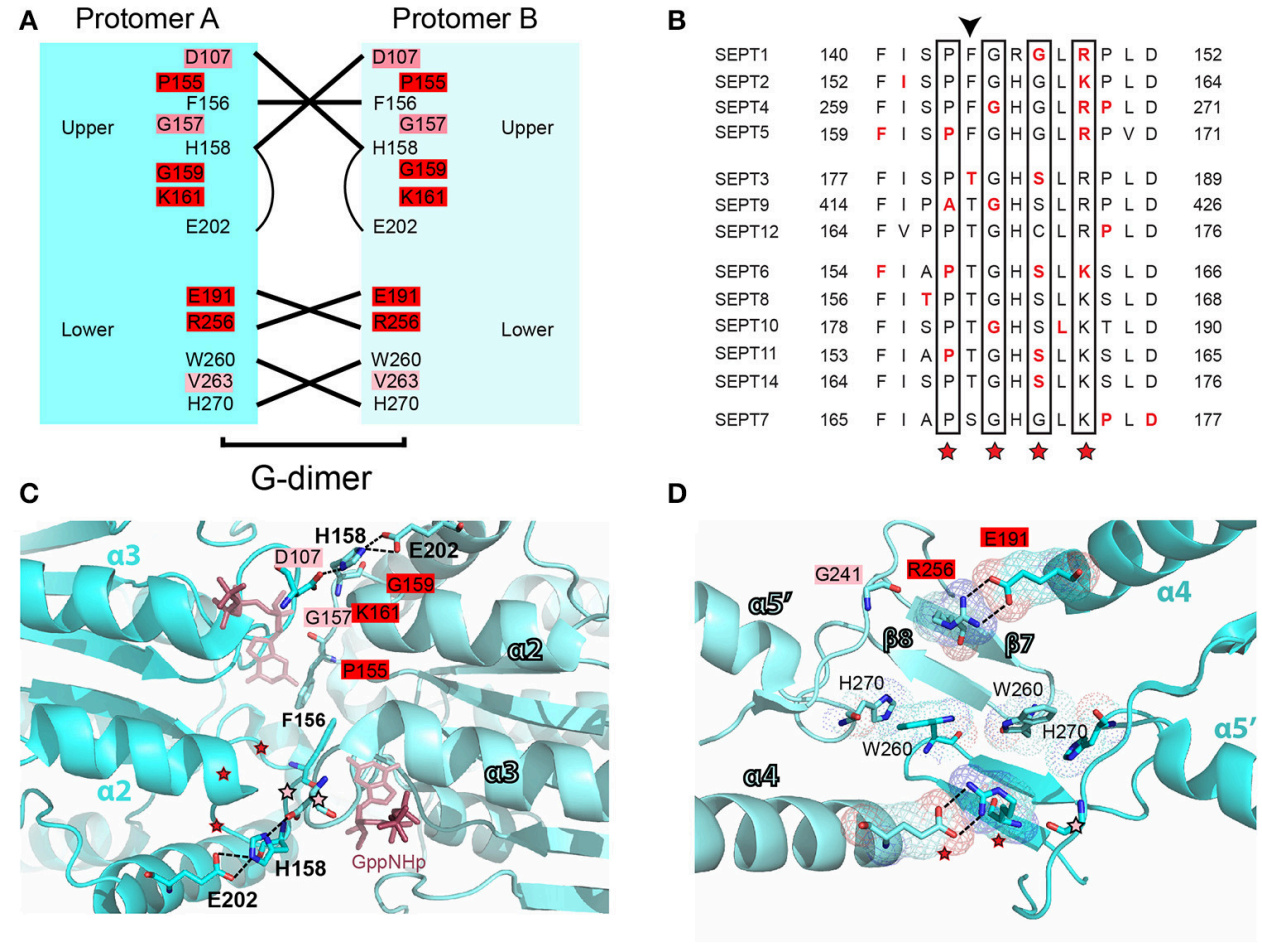
**Mutations in the G-G interface of septin oligomerization. (A)** Open book diagram of the G-G interface of SEPT2 (PDB code: 3FTQ) shows critical residues and interactions (lines) of this interface. Amino acids with 2-3 and 4-5 mutations are highlighted in pink and red, respectively. Open book diagram is a hand-made representation of the approximate position of each amino acid and of their interactions along the z-axis of the crystal structure of SEPT2. **(B)** Sequence alignment of a region that underlies the interactions of the upper G-G interface. Amino acids with missense mutations are shown in red font. The positions of highly conserved amino acids with three or more missense mutations across all septins are outlined with rectangle boxes and red asterisks. Arrowhead points to a phenylalanine residue, whose mutation has been shown to abrogate the dimerization of SEPT2. **(C,D)** Ribbon cartoons depict the upper **(C)** and lower **(D)** G-G interface of SEPT2 (PDB code: 3FTQ). Dotted lines outline interactions between select residues. Amino acids with two or more mutations are highlighted in pink (2-3 mutations) or red (4-5 mutations), and their positions on the opposing protomer are indicated with asterisks of the same color code. Opposing protomers are shown in cyan and aqua marine colors. The non-hydrolyzable GTP analog GppNHp **(C)** is depicted in ruby red. Cartoons were generated in PyMOL. Select bonds (**C**; dashed lines) and electron densities **(D)** were highlighted using the “measure distance,” “mesh,” and “dots” functions of PyMOL.

In the G-G interface, over 25 mutations are clustered on a 12 amino acid patch of the β4/α3 region (Figures 6A,B), and involve amino acids that flank a highly conserved histidine (H158 in SEPT2), which contributes to the tight interactions of the G interface (Weirich et al., 2008; Sirajuddin et al., 2009). Notably, this region includes a phenylanine residue (F156), whose mutation has been shown to disrupt the dimerization of SEPT2 (Sirajuddin et al., 2007). The invariant glycine that precedes H158 is mutated in three different septins (SEPT4, SEPT9, SEPT10) and a highly conserved basic amino acid (arginine or lysine; K161 in SEPT2) is mutated in SEPT6 and all members of the SEPT2 group (Figures 6B,C). A highly invariant proline (P155 in SEPT2) is also mutated in three different septins (Figures 6B,C). In the region outlined by the antiparallel β7 and β8 sheets, the majority of mutations occur in a glutamate (E191 in SEPT2) and an arginine (R256 in SEPT2), which form salt bridges between opposing septin monomers (Figures 6A,D). Mutations in the glutamate residue, which is nearly invariant (aspartate in SEPT14 only), occur in four different septins. R256 is the same invariant arginine of the SUE domain that stacks over the ribose moiety of GTP (see above) and is mutated in five different septins (Figures 5C, 6D). In addition, the preceding arginine (R254) is mutated in three septins of the SEPT2 group. No mutations are identified in the interacting histidine (H270 in SEPT2) and tryptophan (W260 in SEPT2) residues of the β7/β8 sheet region (Figure 6D).

**Figure 7:**
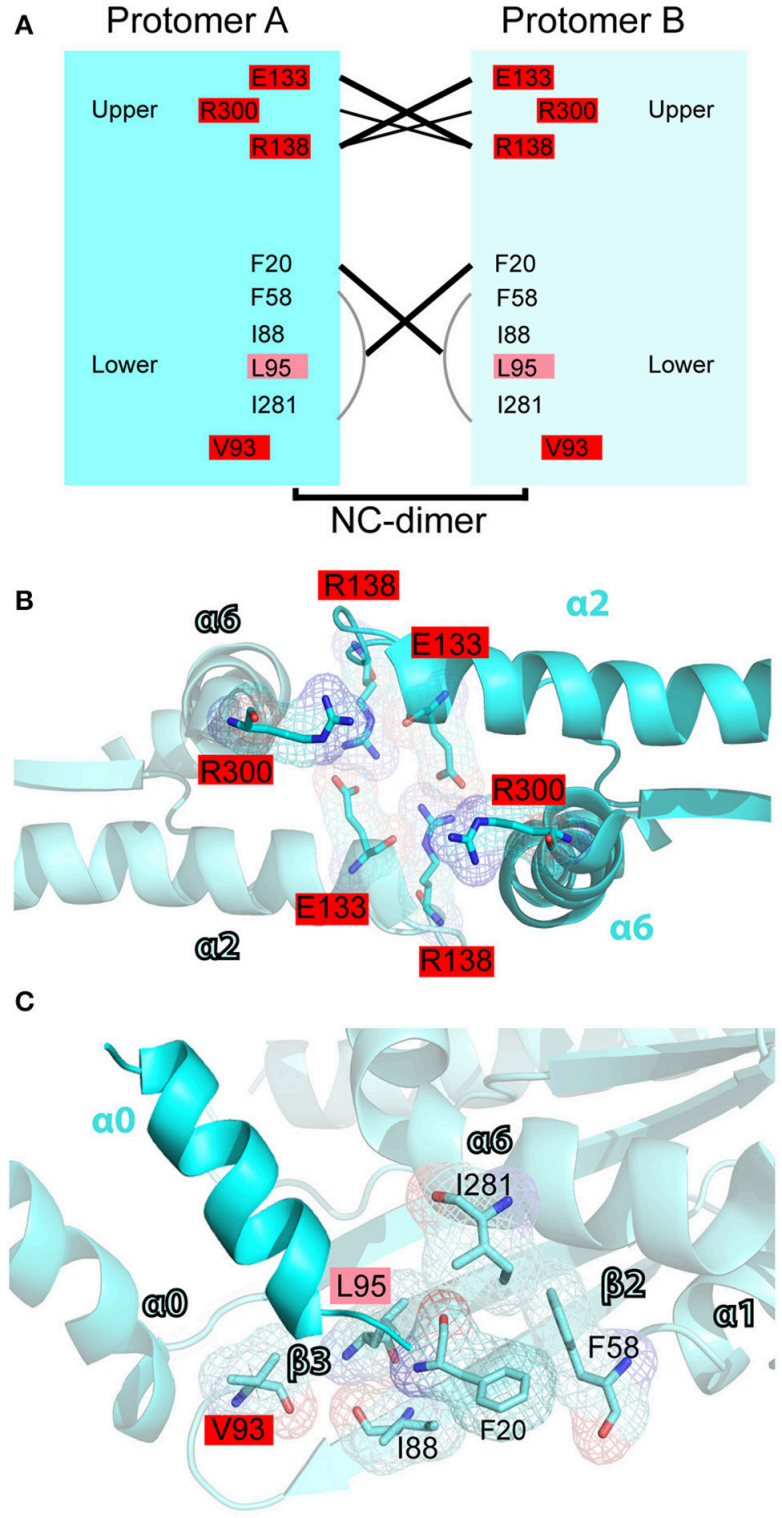
**Mutations in the N-C interface of septin oligomerization. (A)** Open book diagram of the N-C interface of SEPT2 (PDB code: 3FTQ) shows critical residues and interactions (lines) of this interface. Amino acids with 2-3 and 4-5 mutations are highlighted in pink and red, respectively. Open book diagram is a hand-made representation of the approximate position of each amino acid and of their interactions along the z-axis of the crystal structure of SEPT2. **(B,C)** Ribbon cartoons depict the upper **(B)** and lower (D) N-C interface of SEPT2 (PDB code: 3FTQ). Amino acids with two or more mutations are highlighted in pink (2-3 mutations) or red (4-5 mutations). Cartoons were generated in PyMOL and electron densities were highlighted using the “mesh” function of PyMOL.

In the N-C interface (Figure 7A), the majority of septin mutations are clustered on the upper half targeting a major electrostatic interaction between two invariant residues, a glutamate at the end of the α2 helix (E133 in SEPT2) and an arginine in the loop between helix α2 and strand β4 (R138 in SEPT2). Both of these residues are mutated in four different septins (Figures 7A,B). Interestingly, several other mutations are clustered in this region targeting highly conserved arginine residues in the α6 helix of the SUE domain (R280/R293 in SEPT6 and R300 in SEPT2), which may contribute to interactions between opposing monomers (Figure 7B). Each of these arginines is mutated in four different septins. In the lower N-C interface, an invariant valine (V93 in SEPT2) is the most mutated residue; mutations occur in SEPT6, SEPT9, SEPT11 and SEPT14 (Figures 7A,C). No mutations were identified in the valine of the α0 helix (V27 in SEPT2), which interacts hydrophobically with the same valine of the opposing septin monomer; valine is conserved as hydrophobic amino acid (methionine or isoleucine) in all septins. Similarly, no mutations were observed in the highly conserved phenylalanine (F20 in SEPT2; isoleucine in group 3 septins), which forms a hydrophobic pocket with several residues including an invariant phenylalanine (F58 in SEPT2), which is mutated twice in SEPT12, and an isoleucine (I88 in SEPT2) and lysine (L95 in SEPT2), which are mutated in SEPT14 (Figure 7C). Of note, SEPT14 is also mutated in L289 (I281 in SEPT2), which belongs to the SUE domain and projects into the lower N-C interface (Figure 7C).

In summary, the polar interactions between E191 and R256 in the G-G interface and between E133 and R138 in the upper N-C interface emerge as mutational hotspots. Every septin contains at least one mutation that is likely to affect the electrostatic interactions of G-G or N-C interfaces. In addition, the loop region between strand β4 and helix α3, and the SUE are domains that harbor many mutations in conserved amino acids. Four arginine residues of the SUE domain are repeatedly mutated in multiple septins, and one these arginine residues (R256 in SEPT2) is involved in both GTP-binding and septin dimerization. Therefore, mutations in this residue could be rather detrimental for the assembly and function of septins.

## Mutations in the polybasic and coiled-coil domains of septins

The polybasic and coiled coil domains of septins, which are respectively positioned on the N- and C-terminal ends of the GTP-binding domain, are known for their roles in membrane-binding, septin assembly and interaction with binding partners (Zhang et al., 1999; Low and Macara, 2006; de Almeida Marques et al., 2012; Kim et al., 2012). Mutations in these domains could impact the intracellular localization and functions of septins. The phosphoinositide-binding polybasic domain is sparsely mutated, containing only a few substitutions in arginine or lysine residues of SEPT1, SEPT5, SEPT3, and SEPT9. Septins of the SEPT6 group are characterized by a paucity of basic amino acids in their respective regions of the polybasic domain and mutations are minimal. By contrast, the coiled-coil-containing C-terminal tails of the SEPT6 group harbor a significant number of mutations. The C-terminal tails of SEPT14 and SEPT6 contain the most mutations, but very few of these mutations are in amino acids of conserved identity or type. The C-terminal tails of the SEPT2 group of septins, which are shorter, exhibit a similar mutational pattern and SEPT7 has the least number of C-terminal mutations per length of amino acid sequence.

## SEPT9-specific mutations

Among all septins, SEPT9 has been extensively implicated in human cancer. As reviewed above, SEPT9 is linked to the molecular mechanisms of proliferation, angiogenesis, cell invasion and resistance to anti-cancer drugs. SEPT9 contains a unique N-terminal extension with a basic domain, which binds microtubules, actin and the angiogenic HIF1α, as well as an acidic proline-rich domain, which may interact with proteins that contain Src homology 3 (SH3) domains (Golan and Mabjeesh, 2013; Diesenberg et al., 2015; Smith et al., 2015; Bai et al., 2016). In addition, the N-terminal domain of SEPT9 is phosphorylated by the cyclin-dependent kinase 1 (Cdk1) at the onset of mitosis (Estey et al., 2013). Multiple start codons and alternative splice sites give rise to isoforms of variable N-terminal length and sequence (McIlhatton et al., 2001; McDade et al., 2007). These SEPT9 isoforms are likely to possess different binding partners, functions and properties, and their relative levels of expression could affect cell behavior as demonstrated in studies of cell migration (Robertson et al., 2004; Connolly et al., 2011b; Chacko et al., 2012; Dolat et al., 2014b).

**Figure 8:**
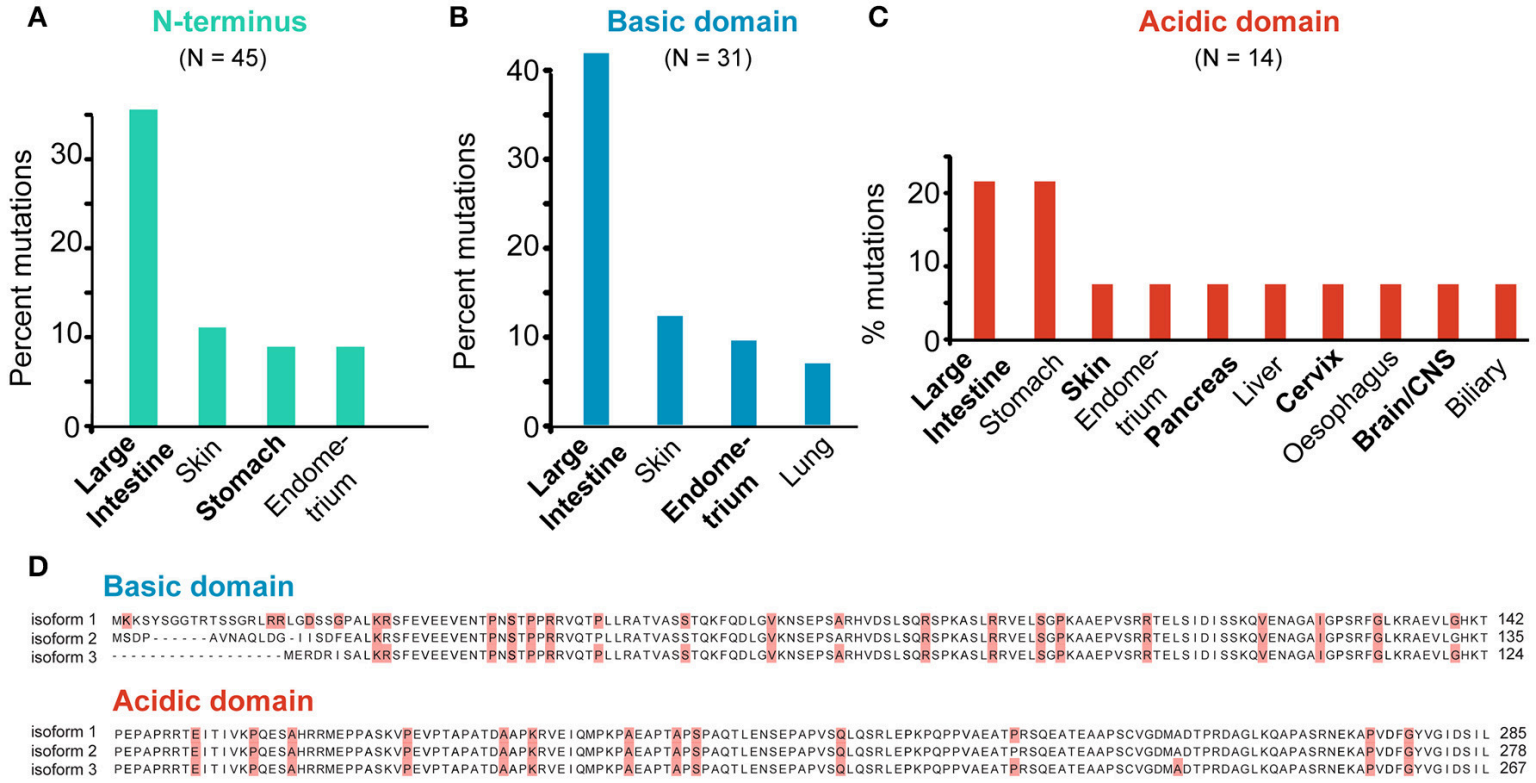
**SEPT9-specific mutations. (A–C)** All missense mutations identified in the N-terminus **(A)** and N-terminal basic **(B)** and acidic **(C)** domains of SEPT9 were grouped per tumor type. Bar graphs show the tumor types which harbor mutations that account for over 5% of the total mutations identified in each domain of SEPT9. **(D)** Sequence alignments of the N-terminal basic and acidic domains of the SEPT9_i1, SEPT9_i2 and SEPT9_i3 isoforms. Missense mutations are highlighted in red.

The majority (62.5%) of all SEPT9 mutations map to the N-terminal domain and nearly 70% of these N-terminal mutations occur in the basic domain. Similar to the full-length SEPT9, nearly a third (~35%) of all N-terminal mutations are found in intestinal cancer samples and approximately 10% are cataloged under skin, stomach or endometrial cancers (Figure 8A). Interestingly, mutations in the N-terminal basic domain of SEPT9 are even more prevalent (~45% of total) in intestinal cancers and mutations in the acidic proline-rich domain of SEPT9 are notably elevated (>20% of total) in stomach cancers (Figures 8B,C).

Previous work has shown that the N-terminal 25 amino acids of the longest SEPT9 isoform (SEPT9_i1) are critical for the activation of HIF1α (Golan and Mabjeesh, 2013). In addition, the basic domain of the SEPT9_i1 (amino acids 1-142) contains the microtubule-binding K/R-R/x-x-E/D motifs (Bai et al., 2013). Missense mutations in five of the N-terminal 25 amino acids of SEPT9_i1 were identified in a variety of cancers (intestine, cervix, lung, skin, liver) and mutations in the basic residues (R91, R107) of two R-R-x-E motifs occur in stomach (R91) and intestinal (R91, R107) cancers (Figure 8D). Interestingly, R107 is mutated in four different patients with intestinal cancer and corresponds to the second arginine of the R-R-x-E motif of SEPT9_i3, which harbors the R88W mutation of hereditary neuralgic amyotrophy (Hannibal et al., 2009). In the acidic proline-rich domain, missense mutations were identified in four proline residues (Figure 8D). Notably, mutations in two amino acids (R45 and P236 of SEPT9_i1) correspond to two of the three PR motifs, which are implicated in the interaction of SEPT9 with the Cbl ubiquitin ligase-interacting protein 85 kD (CIN85), an adaptor protein that promotes the degradation of the epidermal growth factor receptor (EGF-R); SEPT9 prevents binding of CIN85 to ubiquitin ligase (Diesenberg et al., 2015).

## Conclusions and future directions

Since the early 2000s, when mammalian septins began to emerge as a new field of research, there have been major advances in our knowledge of the cellular functions of septins. In parallel, mounting evidence has indicated that septin levels of expression are altered in a variety of cancers. A cause-and-effect relationship between these alterations and tumorigenesis is yet to be established. Septins, however, are involved in mechanisms that promote hallmarks of cancer such as sustained proliferation, resistance to cell death, angiogenesis, cell migration and invasion. Additionally, septins have been implicated in chromosomal instability, resistance to anti-cancer drugs and the induction of tumor growth by the cancer-associated microenvironment. Understanding how abnormalities in septin expression contribute to cancer pathology will benefit from studies that directly test how the up- or down-regulation of certain septins impact tumorigenicity and cancer progression. In these efforts, the use of *in vivo* tumor models will be critical for substantiating septin roles in cancer development.

To date, septin mutations in cancer have not been studied. From the missense mutations that are currently cataloged in COSMIC, the mutational profile of septins appears to be different from the mutations of the evolutionarily related *RAS* oncogenes. Unlike *RAS*, whose mutations exhibit high frequencies and involve select amino acids, septin mutations occur with low frequencies and encompass a variety of residues. While more data are needed to ascertain the lack of “hotspot” amino acids in septins, several missense mutations take place on highly conserved amino acids with key roles in GTP-binding and the G-G and N-C dimerization interfaces. Interestingly, a few mutations occur in amino acids that are linked to male infertily and temperature-sensitive phenotypes in budding yeast.

Future studies are necessary to explore the potential roles and utility of these mutations. Do these mutations result in gain or loss of septin function? Do they have dominant negative or recessive effects? Can they be utilized toward manipulating septin functions *in vitro* and *in vivo*? Are they of diagnostic and/or prognostic value for specific cancers and patients? More importantly, are these mutations a mere consequence of the increased genomic instability that characterizes many cancers or do they play an active role in cancer progression? Answers to these questions will provide more clarity on the role of septins in cancer and may lead to new diagnostic and therapeutic strategies.

## Author contributions

ES conceived the review topic, directed the review of septin mutations and wrote the manuscript. DA performed the meta-analyses of COSMIC data and prepared all figures.

## Funding

This work was supported in part by NIH/NIGMS grant GM097664 to ES.

### Conflict of interest statement

The authors declare that the research was conducted in the absence of any commercial or financial relationships that could be construed as a potential conflict of interest.
